# Geographical and Seasonal Patterns of Geosmin and 2-Methylisoborneol in Environmental Water in Jiangsu Province of China

**DOI:** 10.1155/2014/743924

**Published:** 2014-10-23

**Authors:** Zhen Ding, Shifu Peng, Yuqin Jin, Zhoubin Xuan, Xiaodong Chen, Lihong Yin

**Affiliations:** ^1^School of Public Health, Southeast University, Nanjing, Jiangsu 210009, China; ^2^Department of Environmental and Endemic Diseases Control, Jiangsu Center for Disease Control and Prevention, Nanjing, Jiangsu 210009, China; ^3^Department of Environmental Health, Yangpu Center for Disease Control and Prevention, Shanghai 200090, China

## Abstract

This study was conducted to obtain the basic data of two common odorants—geosmin and 2-methylisoborneol (GSM and 2-MIB)—in environmental water. More specifically, the headspace solid-phase microextraction coupled to gas chromatography mass spectrometry (HS-SPME/GC-MS) was applied to determine the levels of GSM and 2-MIB in water samples, and the samples were collected depending on water sources, conventional treatment processes, and seasons. The significant difference was shown for the 2-MIB levels of source water (*P* < 0.05), the concentrations of GSM and 2-MIB decreased significantly as treatment process of tap water moved forward (*P* < 0.0001), and the significant differences for the levels of GSM and 2-MIB were observed among three sampling periods (*P* < 0.01). The levels of GSM and 2-MIB in all water samples were lower than 10 ng L^−1^, the odor threshold concentration (OTC), and the conventional treatment process plays a significant role in removing odorants in tap water.

## 1. Introduction

Odorants are troublesome in water samples, because they dramatically influence the esthetic quality and consumers' acceptability of drinking water [1, 2]. These odorants are often associated with the metabolites that are produced in the degradation of cyanobacteria, actinomyces, fungi, and blue-green algae [3–5]. The odorants geosmin (GSM) and 2-methylisoborneol (2-MIB) are commonly found in lakes and reservoirs [6, 7], which people can smell their odors in water samples even at the concentration of 10 ng L^−1^ or less, but it would be difficult to identify and quantify these two trace volatile organic compounds (VOCs) [8–12]. As a result, the low threshold of detection can result in consumer complaints about the odors in recreational waters, aquatic products, and tap water, especially during the outbreak period of algal blooms [13], even if some other quality indicators of water, such as turbidity, number of algal cells, and suspended matter, are acceptable.

In recent years, according to the investigation by Chinese Academy of Sciences [14, 15], the results indicated that freshwater lakes were suffering the odor problems in China, especially in Lake Taihu, Lake Chaohu, and Lake Dianchi. These unpleasant odors occurring in these lakes or the aquatic were caused by the volatile secondary metabolites of the algal overgrowth. Therefore, we had conducted this study in 2012, aiming to obtain the basic data of common odorants (GSM and 2-MIB) in environmental waters in Jiangsu province. To be more specific, the headspace solid-phase microextraction coupled to gas chromatography mass spectrometry (HS-SPME/GC-MS) was applied to determine the levels of GSM and 2-MIB in water samples, and this method can be obtained in our early reports [16, 17]. In addition, the water samples were collected in detail as follows: firstly, for water sources of tap water, four kinds of water samples were collected respectively from four cities in Jiangsu, including city A (little lakes as the source), city B (the Changjiang River as source), city C (Lake Taihu as source), and city D (underground water as the source); secondly, for conventional treatment process of tap water, including coagulation and sedimentation, filtration, and disinfection, the water samples were collected respectively from above processes; thirdly, for different seasons of water samples, we collected the samples in three periods in Lake Taihu, including the period when a river is at its normal level (November to December), drought period (February to March), and wet season (July to August) when the algae bloom.

## 2. Experimental

### 2.1. Chemicals and SPME Apparatus

Two common odorants in water, GSM and 2-MIB, and internal standard 2-isobutyl-3-methoxypyrazine (IBMP) were obtained from Sigma-Aldrich (USA) at a concentration of 100 mg L^−1^ in methanol and 1 mg L^−1^ mixed standard solutions of two target compounds in methanol, and all of them were stored in the dark at 4°C. Deionized water was prepared on a water purification system (Gradient A10) supplied by Millipore (Billerica, MA, USA). Sodium chloride (analytical grade, China), which was added to the samples before extraction, was conditioned by heating at 450°C for 4 h before use. SPME apparatus was purchased from Supelco (USA), including fiber 30/50 *μ*m DVB/CAR/PDMS (number 57348-U), fiber holder, 60 mL specialized vials for SPME, sampling stand, magnetic stirrer, and injection catheter (number 57356-U).

### 2.2. SPME Procedures

After putting NaCl and a stir bar in a 60 mL vial, 40 mL of mixed standard solutions for standard curve or 40 mL environmental water samples were added, and 2 *μ*L IBMP (1 mg L^−1^) was added to every sample. The vial was sealed with polytetrafluoroethylene (PTFE) septum cap and placed in a water bath. Several minutes after the temperature was achieved in the vial, the outer needle of fiber was used to penetrate the septum, and the fiber was exposed to the headspace for extraction. After 30 min exposure, the fiber was immediately inserted into GC injection port for desorption.

According to our early study [16, 17], the HS-SPME/GC-MS was applied to determine the levels of GSM and 2-MIB in water samples, and the optimum conditions for HS-SPME were as follows: temperature of extraction and desorption, 65°C and 260°C, respectively; time of extraction and desorption, 30 min and 5 min, respectively; ionic strength, 25% (w/v); rotation speed, 600 rpm; solution pH, 5.0.

### 2.3. Gas Chromatography-Mass Spectrometry

A Varian 300 GC/MS/MS (Varian Inc., CA, USA) with ion trap and mass spectrometer was obtained with a Varian VF-5 MS capillarity column (30 m × 0.25 mm × 0.5 *μ*m). The temperature of the injector was 260°C adjusted to splitless mode. The carrier gas was helium at a flow of 1 mL min^−1^. The temperature of the oven started at 40°C and was held for 5 min. Then the temperature was 8°C min^−1^ to achieve 160°C (total time 20 min) followed by 20°C min^−1^ to achieve 260°C (total time 25 min). The electron impact (EI)-MS conditions were as follows: ion-source temperature, 230°C; MS transfer line temperature, 250°C; solvent delay time, 5 min; ionizing voltage, 70 eV. The mass spectrogram in full scan mode was obtained at the* m*/*z* range of 80–200 u. Based on the MS scan function (SIM mode), the process was divided into three main segments. The method of internal standard was applied to construct calibration curve and determine concentrations of 2-MIB and GSM in water.

### 2.4. Water Samples Collection

#### 2.4.1. Water Samples from Different Cities

Based on different sources for tap water, four representative cities were chosen to collect water samples, including city A (little lakes as the source), city B (the Changjiang River as source), city C (Lake Taihu as source), and city D (underground water as the source). Five water works were selected from each city, and raw water, output water, and end water (for consumers) were obtained respectively from each water work in drought period—February to March—by manual sampling, meaning 15 samples in total in one city. Water samples from the water works were analyzed by the proposed method, which were kept in 350 mL sample vials with PTFE-faced silicone septum and stored at 4°C before analysis. The average length of time between sampling and analysis was 24 hours.

#### 2.4.2. Water Samples from Different Processes of Tap Water Treatment

**Figure 1:**
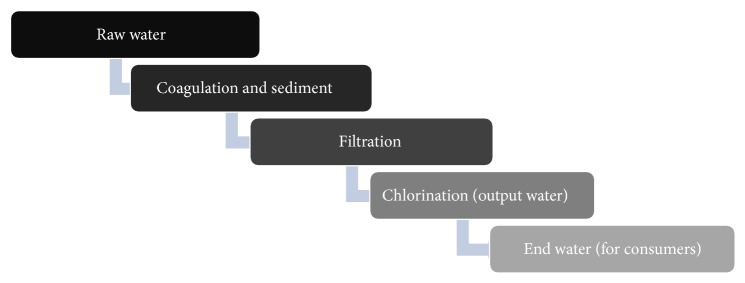
The conventional treatment process for tap water.

The conventional treatment of tap water includes four processes, as raw water, coagulation and sedimentation, filtration, and disinfection, and end water was provided for consumers by pipe network as shown in Figure 1. The five water works were selected from city B (shorter distance to laboratory than other three), and one sample was obtained from each process, that is to say, 4 samples in total in one water work.

#### 2.4.3. Water Samples from Different Seasons

For different seasons of water samples, we collected the samples in three periods in Lake Taihu, including the period when a river is at its normal level (November to December), drought period (February to March), and wet season (July to August) when the algae bloom. In addition, Lake Taihu was used as sampling point, because the algal overgrowth appears every summer in this lake, and the consumers complain that the uncomfortable odors exist in their drinking water during the outbreak period of algal blooms. The five water works were selected from city C, and raw water, output water, and the end water were obtained respectively from each water work, 15 samples in total for one period.

### 2.5. Statistical Methods

The analysis of variance (ANOVA) was applied to the present work, aiming to obtain the significant differences when comparing average values in the two or more groups. More specifically, two-way ANOVA was applied to compare the GSM and 2-MIB levels in four cities (Tables 1 and 2), while one-way ANOVA was applied in water samples from different processes of tap water treatment and different seasons (Tables 3 and 4).

## 3. Results and Discussion

According to Tables 1 and 2, the significant differences of concentration cannot be observed for both GSM and 2-MIB among the four cities (*P* > 0.05), excepting the 2-MIB levels of raw water (*P* < 0.01). More specifically, the 2-MIB level of raw water in city D was lower than that in three other cities (*P* < 0.01), and the most potential reason could be that the soil or rocks work as natural barrier or filter, keeping the larger particles permeating into raw water. As a result, the level of 2-MIB may be lower in city D. However, there are no significant differences for the levels of GSM among four cities; in other words, the lower 2-MIB levels of raw water in city D never necessarily equal the lower levels of GSM in city D similarly. It can be explained that the levels of two odorants were associated with multiple factors, including the species of algae, acidic condition of environment, rainfalls, and light density [18]. For example, growth and production of 2-MIB was characterized in the cyanobacterium* Phormidium* sp. [19] but was not responsible for GSM. Another example is that the actinomycetes can cause the odorants, but the total colony counts did not necessarily indicate the direct role of actinomycetes in odor problems in environmental water, because their activities would be restricted by other environmental factors [20]. Consequently, the lower 2-MIB occurred in city D, but GSM did not, even though they shared the same water source.

In addition, the decreased levels of GSM or 2-MIB were observed among the raw water, output water, and end water (*P* < 0.05), as implied in Table 4. Because both of GSM and 2-MIB were volatile organic chemicals (VOCs), the concentrations would decrease in the water transferred from water works to destination, and similar results are obtained by early reports in China [21]. Meanwhile, the concentration of total residual chlorine in pipe network would necessarily reduce the levels of GSM and 2-MIB [22, 23]. But, however, an experimental error (10–70%) may be observed due to the presence of free residual chlorine, and this error can decline after dechlorination (less than 10%) [22, 24]. Consequently, the average values of two odorants in Table 3 may increase from filtration to chlorination.

Based on Table 3, it is indicated that the concentration of GSM and 2-MIB decreased significantly as treatment process moved forward (*P* < 0.0001). The reasons can be as follows: firstly, after the processes of coagulation, sedimentation, and filtration, larger particles had been removed, such as algae, silt, and other plankton, which can produce the musty and earthy odorant in tap water, and the same result was implied in early study [25]. The last process, chlorination, can kill or remove main cyanobacteria and actinomyces by oxidation [26, 27]. And also some reports indicated that the presence of chlorine would substantially reduce the observed GSM and 2-MIB concentrations, and its impact was larger for lower organic compound concentrations, under higher residual chlorine conditions [22]. Consequently, the lower levels of GSM and 2-MIB would appear in output water.

The water samples of different seasons were collected from city C, the Lake Taihu of which was used as source water and blue-green algae blooming in summer in this lake. According to Table 4, the significant differences were observed for the levels of GSM and 2-MIB among the three periods (*P* < 0.01); in other words, the GSM and 2-MIB levels of raw water were higher in period of wet water than corresponding levels in other two periods. The most important reason was the algae blooming and reservoir's eutrophication in summer, resulting in higher concentrations of odorants, while the algae or cyanobacteria were dead or inactive in other periods. Moreover, the concentrations were found to correlate with corresponding air and water temperatures, and the average water temperature was 12, 16, and 27°C for normal level, drought, and wet period, respectively. The concentrations of odorants followed a trend of higher levels during the warm seasons [28–30]. Consequently, the levels in wet season (in summer) were higher than those in other two periods. Also, the results implied that levels of odorants were approximately equal between the periods of normal level and drought.

## 4. Conclusion

The levels of GSM and 2-MIB in water samples were less than 10 ng L^−1^, the odor threshold concentration (OTC) [12, 31]. And the conventional treatment process of tap water plays a significant role in removing common odorants in running water for these source waters. In particular the largest decrease in concentrations occurs after sedimentation suggesting that removal of GSM and MIB in intact algae cells is important.

## Figures and Tables

**Table 1:** The statistical analysis of GSM levels in four cities (*n* = 60).

City	Raw water (ng L^−1^)	Output water (ng L^−1^)	End water (ng L^−1^)	Comparative analysis in one city	
				*F* value	*P* value
A	2.27 ± 1.45	0.22 ± 0.1	0.09 ± 0.04	7.07	0.0143
B	0.72 ± 0.17	0.21 ± 0.11	0.14 ± 0.09	26.08	0.0002
C	3.55 ± 0.66	0.56 ± 0.67	0.06 ± 0.02	44.04	<0.001
D	0.73 ± 0.18	0.18 ± 0.17	0.08 ± 0.02	26.18	0.0001

Comparing among four cities					
*F*	0.84	0.84	1.64		
*P*	0.50	0.50	0.24		

**Table 2:** The statistical analysis of 2-MIB levels in four cities (*n* = 60).

City	Raw water (ng L^−1^)	Output water (ng L^−1^)	End water (ng L^−1^)	Comparative analysis in one city	
				*F* value	*P* value
A	7.56 ± 3.38	1.50 ± 0.57	0.46 ± 0.33	10.24	0.0062
B	7.59 ± 2.85	1.64 ± 1.27	0.06 ± 0.04	11.16	0.0156
C	6.52 ± 2.71	1.46 ± 0.70	0.04 ± 0.01	17.31	0.0004
D	1.30 ± 1.02	0.36 ± 0.25	0.06 ± 0.04	2.43	0.1575

Comparing among four cities					
*F*	6.46	1.91	2.56		
*P*	0.0045	0.19	0.17		

**Table 3:** Water samples from different processes of tap water treatment (*n* = 20).

Compound	Raw water (ng L^−1^)	Coagulation and sediment (ng L^−1^)	Filtration (ng L^−1^)	Chlorination (ng L^−1^)	*F*	*P*
GSM	0.72 ± 0.17	0.56 ± 0.22	0.12 ± 0.03	0.21 ± 0.11	15.65	<0.0001
2-MIB	7.58 ± 2.85	1.96 ± 0.45	0.75 ± 0.86	1.63 ± 1.28	16.69	<0.0001

**Table 4:** Water samples from different seasons (*n* = 45).

Compound	Water type	Normal level^a^ (ng L^−1^)	Drought period^b^ (ng L^−1^)	Wet season^c^ (ng L^−1^)	*F*	*P*
GSM	Raw water	4.25 ± 2.56	3.55 ± 0.66	7.61 ± 1.70	7.07	0.01
	Output water	1.06 ± 0.41	0.56 ± 0.67	1.23 ± 1.03	0.85	0.46
	End water	0.18 ± 0.16	0.06 ± 0.02	0.04 ± 0.00	1.71	0.25

2-MIB	Raw water	5.12 ± 2.16	6.52 ± 2.71	10.17 ± 1.96	6.43	0.01
	Output water	1.02 ± 0.33	1.46 ± 0.70	1.15 ± 0.49	0.89	0.43
	End water	0.63 ± 0.63	0.04 ± 0.01	0.21 ± 0.09	1.08	0.38

Note: a, b, and c represent algal cell count in their raw waters, and their values were 6.21 m L^−1^, 3.34, and 11.42, respectively.

## References

[1] Mackey E. D., Baribeau H., Crozes G. F., Suffet I. H., Piriou P. (2004). Public thresholds for chlorinous flavors in U.S. tap water. *Water Science and Technology*.

[2] Freuze I., Brosillon S., Herman D., Laplanche A., Démocrate C., Cavard J. (2004). Odorous products of the chlorination of phenylalanine in water: formation, evolution, and quantification. *Environmental Science and Technology*.

[3] Izaguirre G., Taylor W. D. (2004). A guide to geosmin- and MIB-producing cyanobacteria in the United States. *Water Science and Technology*.

[4] Gerber N. N. (1967). Geosmin an earthy-smelling substance isolated from actinomycetes. *Biotechnology and Bioengineering*.

[5] Gerber N. N., Lechevalier H. A. (1965). Geosmin, an earthly-smelling substance isolated from actinomycetes. *Applied Microbiology*.

[6] Peter A., Köster O., Schildknecht A., von Gunten U. (2009). Occurrence of dissolved and particle-bound taste and odor compounds in Swiss lake waters. *Water Research*.

[7] Schrader K. K., Rubio S. A., Piedrahita R. H., Rimando A. M. (2005). Geosmin and 2-methylisoborneol cause off-flavors in cultured largemouth bass and white sturgeon reared in recirculating-water systems. *North American Journal of Aquaculture*.

[8] Salemi A., Lacorte S., Bagheri H., Barceló D. (2006). Automated trace determination of earthy-musty odorous compounds in water samples by on-line purge-and-trap-gas chromatography-mass spectrometry. *Journal of Chromatography A*.

[9] Watson S. B., Ridal J., Zaitlin B., Lo A. (2003). Odours from pulp mill effluent treatment ponds: the origin of significant levels of geosmin and 2-methylisoborneol (MIB). *Chemosphere*.

[10] Uwins H. K., Teasdale P., Stratton H. (2007). A case study investigating the occurrence of geosmin and 2-methylisoborneol (MIB) in the surface waters of the Hinze Dam, Gold Coast, Australia. *Water Science and Technology*.

[11] Benanou D., Acobas F., Deroubin M. R., David F., Sandra P. (2003). Analysis of off-flavors in the aquatic environment by stir bar sorptive extraction-thermal desorption-capillary GC/MS/olfactometry. *Analytical and Bioanalytical Chemistry*.

[12] Mallevialle J. (1987). *Identification and Treatment of Tastes and Odors in Drinking Water*.

[13] Davies J.-M., Roxborough M., Mazumder A. (2004). Origins and implications of drinking water odours in lakes and reservoirs of British Columbia, Canada. *Water Research*.

[14] Xu Y., Ni W., Wu W. Z. (1999). Study on aquatic off-flavors in eutrophic Donghu Lake. *Acta Ecologica Sinica*.

[15] Li L., Chen W., Song L. R. Extermination of earthy/musty odorous metabolites in lakes and ponds in China.

[16] Peng S., Ding Z., Xia W., Zheng H., Xia Y., Chen X. (2013). Orthogonal design study on factors affecting the determination of common odors in water samples by headspace solid-phase microextraction coupled to GC/MS. *Journal of Analytical Methods in Chemistry*.

[17] Ding Z., Peng S., Xia W., Zheng H., Chen X., Yin L. (2014). Analysis of five earthy-musty odorants in environmental water by HS-SPME/GC-MS. *International Journal of Analytical Chemistry*.

[18] Zhong F., Gao Y., Yu T., Zhang Y., Xu D., Xiao E., He F., Zhou Q., Wu Z. (2011). The management of undesirable cyanobacteria blooms in channel catfish ponds using a constructed wetland: contribution to the control of off-flavor occurrences. *Water Research*.

[19] Zimmerman W. J., Soliman C. M., Rosen B. H. (1995). Growth and 2-methylisoborneol production by the cyanobacterium Phormidium LM689. *Water Science and Technology*.

[20] Sivonen K. (1982). Factors influencing odour production by actinomycetes. *Hydrobiologia*.

[21] Fang F. F., Yu J. W., Yang M. (2009). Determination of dimethyl trisulfide in water by headspace solid-phase micro-extraction coupled with gas chromatography with mass spectrometry,. *China Water and Waste Water*.

[22] Lin T.-F., Liu C.-L., Yang F.-C., Hung H.-W. (2003). Effect of residual chlorine on the analysis of geosmin, 2-MIB and MTBE in drinking water using the SPME technique. *Water Research*.

[23] Proulx F., Rodriguez M. J., Sérodes J. B., Bouchard C. (2012). Spatio-temporal variability of tastes and odors of drinking water within a distribution system. *Journal of Environmental Management*.

[24] Oestman E., Schweitzer L., Tomboulian P., Corado A., Suffet I. H. (2004). Effects of chlorine and chloramines on earthy and musty odors in drinking water. *Water Science and Technology*.

[25] Pan R. P., Deng H. P., Xu H. (2010). Treatment of micro-polluted water resources by powered activated carbon-ultrafiltration membrane process. *Technology of Water Treatment*.

[26] Bai X.-H., Zhang M.-D., Jia C.-S. (2011). Migration of main odorous compounds in a water supply system with Huangpu River as raw water in Shanghai. *Huan Jing Ke Xue*.

[27] Jung S.-W., Baek K.-H., Yu M.-J. (2004). Treatment of taste and odor material by oxidation adsorption. *Water Science and Technology*.

[28] Tung S.-C., Lin T.-F., Yang F.-C., Liu C.-L. (2008). Seasonal change and correlation with environmental parameters for 2-MIB in Feng-Shen Reservoir, Taiwan. *Environmental Monitoring and Assessment*.

[29] Westerhoff P., Rodriguez-Hernandez M., Baker L., Sommerfeld M. (2005). Seasonal occurrence and degradation of 2-methylisoborneol in water supply reservoirs. *Water Research*.

[30] Ho L., Tang T., Monis P. T., Hoefel D. (2012). Biodegradation of multiple cyanobacterial metabolites in drinking water supplies. *Chemosphere*.

[31] Young W. F., Horth H., Crane R., Ogden T., Arnott M. (1996). Taste and odour threshold concentrations of potential potable water contaminants. *Water Research*.

